# Experimental Investigation and Modeling of Surface Roughness in BTA Deep Hole Drilling with Vibration Assisted

**DOI:** 10.3390/ma18010056

**Published:** 2024-12-26

**Authors:** Xubo Li, Chuanmiao Zhai, Canjun Wang, Ruiqin Wu, Cunqiang Zang, Shihao Zhang, Bian Guo, Yuewen Su

**Affiliations:** 1 College of Mechanical Engineering, Baoji University of Arts and Sciences, Baoji 721016, China; 2 Zhejiang Sankai Mechanical and Electrical Co., Ltd., Taizhou 317511, China; 3 School of Mechanical and Precision Instrument Engineering, Xi’an University of Technology, Xi’an 710048, China

**Keywords:** BTA deep hole drilling, vibration drilling, surface roughness, process parameter optimization

## Abstract

The surface roughness of hole machining greatly influences the mechanical properties of parts, such as early fatigue failure and corrosion resistance. The boring and trepanning association (BTA) deep hole drilling with axial vibration assistance is a compound machining process of the tool cutting and the guide block extrusion. At the same time, the surface of the hole wall is also ironed by the axial large amplitude and low-frequency vibration of the guide block. The surface-forming mechanism is very complicated, making it difficult to obtain an effective theoretical analytical model of the surface roughness of the hole wall through kinematic analysis. In order to achieve accurate prediction of the surface quality of the hole wall, the chip-breaking mechanism and the hole wall formation mode of BTA deep hole vibration drilling were analyzed. The influence of drilling spindle speed, feed, amplitude, and vibration frequency on the surface roughness of the hole wall during BTA deep hole vibration drilling was illustrated by a single-factor experiment. A four-factor and three-level test scheme was designed by using the Box–Behnken design (BBD) experimental design method. A surface roughness prediction model for hole wall machining was established based on the response surface methodology. The accuracy of the prediction model was analyzed through ANOVA, and the complex correlation coefficient of the model was 0.9948, indicating that the prediction model can better reflect the mapping relationship between vibration drilling parameters and surface roughness. After optimization analysis and experimental verification, the obtained vibration drilling parameters can achieve smaller surface roughness. The error between the predicted value of the model and the experimental measurement value is 8.65%. The established prediction model is reliable and can accurately predict the surface roughness of the hole wall of BTA deep hole axial vibration drilling, providing a theoretical basis for the surface quality control of the machining hole wall. It can be applied to process optimization in practical production.

## 1. Introduction

Precision and efficient deep-hole machining technology is today an urgent requirement for the precision machining of deep-hole parts in high-tech industries such as military manufacturing, new energy equipment manufacturing, and aerospace [1,2,3]. BTA deep hole drilling technology is an internal chip removal processing method. Compared with gun drilling, BTA deep hole drilling uses a higher stiffness of the round tube as the transmission power chip removal tool, which allows for a larger feed and prevents chips from being discharged from the circular tube to avoid scratching the machined hole wall. In addition, the guide block with a circular distribution of the BTA drill body can balance the cutting force of the cutter teeth and iron the hole wall to achieve the self-guidance of drilling and improve the hole machining quality [4,5,6].

Due to the limitation of chip removal channel area, chip breaking and removal have always been difficult processes in deep hole drilling with internal chip removal, especially in the deep hole processing of high-strength low-carbon alloy steel, such as nuclear power tube sheets. Due to the high strength, toughness, and material plasticity, chip breaking is difficult; chip blocking and tool breakage often occur, directly affecting the efficiency of deep hole drilling [7,8].

Through the auxiliary axial vibration at the end of the drill pipe, the size of chips can be controlled to improve cutting and chip discharge efficiency. BTA deep hole vibration drilling technology is an effective method to solve the problem of precision deep hole machining of difficult materials, and its technological effect has been accepted. However, not all vibration drilling results in good machining results [9]. The process of vibration drilling is always accompanied by separation cutting, variable cutting angle, variable cutting speed, and cutting thickening, which makes the dynamic characteristics of the tool system very complicated in the process of vibration drilling [10].

In the research of BTA deep hole vibration drilling, Guba et al. [11] studied the positive basalt-assisted hole drilling with low frequency and large vibration amplitude, leading to the interruption of the cutting motion, indicating that the cutting heat generated by vibration drilling is much lower than that of normal production drilling, and the friction generated by drilling is smaller, which can effectively avoid the accumulation and adhesion of air edge materials. Based on the oblique cutting theory, Li et al. [12] established the BTA deep hole vibration drilling analytical model considering the scale effect of dynamic cutting thickness and the rule of drilling radius distribution and revealed the influence of vibration drilling process parameters on the drilling force. Shi et al. [13] used finite element simulation to report the temperature distribution changes of bits and workpieces under the ordinary drilling and vibration drilling of nickel-based alloys. The results show that BTA deep hole vibration drilling effectively reduces the cutting temperature and improves the wear resistance of the tool. Jiao et al. [14] developed a low-frequency vibration-assisted drilling device using a ring hinge as an elastic mechanism. The axial low-frequency vibration can realize a constant frequency ratio and amplitude stepless adjustment, which can reduce the drilling temperature by 44%. Han et al. [15] studied the influence of drilling process parameters on the drilling temperature and chip morphology and applied low-frequency vibration-assisted drilling to medical bone hole processing. Erfani et al. [16] explored the influence of low-frequency vibration-assisted drilling on chip morphology and surface roughness and showed that vibration drilling can effectively reduce the surface roughness of the machining hole wall. However, the above studies have not clearly defined the mapping relationship between vibration drilling parameters and surface quality.

Surface quality usually directly affects the physical, chemical, and mechanical properties of the workpiece, such as frictional properties, wear resistance, lubrication ability, etc. The surface quality of the hole wall in the deep hole processing of the tube plate of the nuclear power steam generator directly affects the contact fit degree, corrosion resistance, and radiation protection characteristics of the heat exchange tube bundle and the hole wall, and then affects the heat exchange efficiency and use safety of the nuclear power equipment [17,18]. Surface roughness is an important index to evaluate surface quality, so it has been selected as a key technical requirement for parts production. Although there are various evaluation indexes for surface roughness, the contour arithmetic mean deviation *R*a is widely used as an important technical index to characterize surface roughness [19,20,21].

Many scholars have tried to control the surface roughness of the hole wall by optimizing the deep hole drilling process. Feng et al. [22] used BTA deep hole tools with different structural parameters to perform deep hole drilling experiments on TA10 materials and optimized the deep hole machining process parameters based on multiple objectives (such as chip shape, tool wear, hole axis deflection, and hole surface roughness). In order to improve the material removal rate and minimize roundness deviation, Chandar et al. [23] applied the grey wolf optimization (GWO) algorithm to optimize the process parameters of deep hole drilling. Pham et al. [24] established a surface roughness prediction model for milling AISI H11 steel with minimal lubrication and applied the mathematical model to the optimization of milling parameters. Kouahla et al. [25] used PVD-coated carbide cutting tools to dry turn Inconel718 alloy and established a prediction model of surface roughness. Zhang et al. [26] explored the surface forming mechanism of BTA deep hole machining from a microscopic level and studied the surface roughness and surface microstructure through experiments. The results showed that the improvement of the hole surface integrity depended on the balance between drilling speed and feed rate. Woon et al. [27] explained the influence of the guide block on the surface quality of the hole wall in deep hole drilling, and the results indicated that the extrusion of the guide strip on the hole wall would greatly affect the surface quality. Motorcu et al. [28] studied the effects of spindle speed, feed, and tool angle on the surface roughness during the drilling of nickel-based alloys. Some scholars also use the response surface methodology combined with other methods to establish surface roughness prediction models. Ramesh et al. [29] obtained sample data such as tool vibration, tool wear, and cutting temperature through experiments and applied them to the training of an artificial neural network to develop a prediction model of drilling process feature parameters based on an artificial neural network. Cicek et al. [30] demonstrated the effects of low-temperature treatment and drilling parameters on surface and hole quality. Analysis of variance was used to determine the most significant control factors for surface roughness and roundness errors. Balaji et al. [31] exposed the effects of spindle speed, tool helix angle, and feed on surface roughness and tool wear through the orthogonal experiment of twisting drill for machining titanium alloy and established the prediction model of surface roughness and tool wear through the response surface method. In actual processing, surface roughness constantly changes and often increases due to tool vibration, tool wear, and plastic deformation of the workpiece material. Therefore, an effective method for stabilizing surface roughness is needed to achieve better surface performance of workpieces. A high-precision surface roughness prediction model is not only the basis for controlling and stabilizing surface roughness but also can avoid high costs and long experimental measurement processes.

However, due to the complexity of the BTA deep hole vibration drilling mechanism, the tool system is affected by many factors such as drill tube stiffness, auxiliary support, guide sleeve, and cutting fluid flow force during the BTA deep hole drilling. The influence of each factor on the surface quality of the hole is not a simple superposition but the result of mutual influence and mutual coupling, which cannot be described by explicit mathematical expression. There is currently no report on the prediction model of the surface roughness of the hole wall for deep hole vibration drilling. In order to achieve accurate prediction of the surface quality of the hole wall, the chip breaking mechanism and hole wall formation method of BTA deep hole vibration drilling were analyzed. The influence of process parameters such as spindle speed, feed rate, amplitude, and vibration frequency on the surface roughness of the hole during the BTA deep hole vibration drilling of nuclear tube plate material was studied through single-factor experiments. A four-factor, three-level experimental scheme was designed by using the BBD experimental method, and a surface roughness prediction model of machining hole wall was established based on the response surface methodology. Study the significance of the influence of various factors and their interactions on surface roughness in the prediction model through ANOVA analysis. Based on the established second-order response model of surface roughness, the machining process parameters are optimized by using the satisfaction function method. Provide a theoretical basis for the surface quality control of the BTA deep hole vibration drilling hole wall, which can be applied to process optimization in practical production.

## 2. Vibration Drilling Mechanism

**Figure 1:**
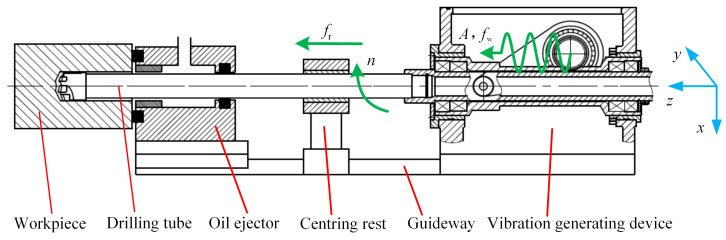
Schematic diagram of BTA axial vibration drilling system.

The working principle of BTA deep hole drilling with axial vibration assistance is shown in Figure 1. In the drilling, the spindle motor drives the drill tube to rotate at a constant speed *n*, the spindle screw motor drives the vibration device to move in the direction of the *z* axis with a constant feed *f*_r_, and the drill pipe and the eccentric spindle, eccentric sleeve, and connecting rod of the vibration-generating device constitute a set of crank slider mechanisms. Under the action of the vibration motor, the eccentric shaft rotates and drives the drill tube to make axial reciprocating movement with constant vibration amplitude *A* and vibration frequency *f*_w_. By changing the angle between the eccentric axis and the center of the eccentric sleeve, the eccentricity is changed and the amplitude is adjusted. The vibration frequency can be adjusted by changing the speed of the vibration motor.

The BTA deep hole drill bit is connected to a hollow drilling tube with a trapezoidal thread, and the end of the drill tube is connected to a vibration source. The cutting oil enters through the high-pressure oil inlet on the oil ejector, and under the action of the oil pump, the high-pressure oil flows into the drilling area from the gap between the workpiece hole wall and the outer surface of the drilling tube, and the chips generated during the machining are flushed into the inner cavity from the two sizes of the chip discharge port of the drilling tube, and finally discharged to the chip collecting box through the inner cavity of the drilling tube. The whole process can realize the cooling of the BTA tool, the lubrication of the cutter teeth, and the contact surface of the guide block, so that the whole drilling process maintains a stable and efficient processing state. The BTA deep hole drilling process is an internal chip removal process, which avoids direct contact between chips and the hole wall and ensures the machining accuracy of the hole.

According to the kinematic relationship in Figure 1, it can be seen that the axial vibration displacement of the drilling tube is a harmonic curve, so the vibration displacement equation of the drill along the axis is defined as

(1) $$z_{c}\left(t\right)=A\sin\left(2\pi f_{w}t\right)$$

where, *A* stands for amplitude, unit mm; *f*_w_ indicates the vibration frequency, unit Hz.

The displacement of any point on the tool along the axis is the compound superposition effect of feed motion and vibration displacement, and its motion equation can be expressed as

(2) $$z\left(t\right)=A\sin\left(2\pi f_{w}t\right)+f_{r}\left(\frac{n}{60}\right)t$$

where, *n* represents spindle speed, unit r/min; *f*_r_ indicates the quantity of feed in mm/r.

In the drilling process, the drill rotates under the drive of the drilling tube, and the relationship between the rotation angle *θ* and the time *t* can be given as

(3) $$\theta (t)=2\pi \left(\frac{n}{60}\right)\cdot t$$

Substituting Equation (3) into Equation (2), it can be obtained that the motion equation of drill axial displacement with angle, as

(4) $$z\left(\theta \right)=A\sin\left(\frac{60f_{w}}{n}\cdot \theta \right)+\frac{f_{r}\theta }{2\pi }=A\sin\left(\omega_{f}\cdot \theta \right)+\frac{f_{r}\theta }{2\pi }$$

where, *ω_f_* represents the frequency rotation ratio, and its value *ω_f_* = 60*f*_w_/*n* = *N* + *i*, *N* represents the value of the integer part of the frequency rotation ratio, and *i* represents the value of the decimal part.

BTA deep hole drilling is single-edge cutting; the drill rotates once a cycle. According to Equation (4), the axial motion trajectories of two periods adjacent to any point on the drill can be determined from

(5) $$z_{1}\left(\theta \right)=A\sin\left(\omega_{f}\cdot \theta \right)+\frac{f_{r}\theta }{2\pi }$$

(6) $$z_{2}\left(\theta \right)=A\sin\left[\omega_{f}\cdot \left(\theta +2\pi \right)\right]+\frac{f_{r}}{2\pi }\left(\theta +2\pi \right)$$

In the actual drilling process, a complete chip generation may go through several rotation cycles. Assuming that chips are generated after *k* rotation cycles, the axial motion trajectory of the bit in the *k* cycle can be given as

(7) $$z_{k}\left(\theta \right)=A\sin\left[\omega_{f}\cdot \left(\theta +\left(k-1\right)\cdot 2\pi \right)\right]+\frac{f_{r}}{2\pi }\left(\theta +\left(k-1\right)\cdot 2\pi \right)$$

The dynamic cutting thickness of the tool teeth can be expressed as the axial distance between the axial motion trajectory corresponding to the last rotation cycle of the chip formation and its adjacent motion trajectory [12]

(8) $$h\left(\theta \right)=z_{k}\left(\theta \right)-\max\left[z_{1}\left(\theta \right),z_{2}\left(\theta \right),\ldots z_{k-1}\left(\theta \right)\right]$$

After drilling parameters are given during drilling, in order to determine the value of *k*. It can be assumed that the last axial motion path *z_k_*(*θ*) forming chips does not intersect with the first axial motion path *z*_1_(*θ*) [12].

(9) $$z_{k}\left(\theta \right)-z_{1}\left(\theta \right)={\left\{kf_{r}+2A\cos\left[\omega_{f}\left(\theta +k\pi \right)\right]\sin\left(\omega_{f}\cdot k\pi \right)\right\}}_{\min}\geq 0$$

Further calculation, it can be evaluated by

(10) $$z_{k}\left(\theta \right)-z_{1}\left(\theta \right)=\frac{kf_{r}}{2A\left|\sin\left(\omega_{f}\cdot k\pi \right)\right|}\geq 1$$

Therefore, the minimum value of *k* satisfying Equation (10) is the corresponding last axial motion track line.

In the process of vibration drilling, if the axial motion track lines corresponding to each rotation period of the drill are always disjoint, that is, the dynamic cutting thickness is always greater than zero.

(11) $$\frac{2A}{f_{r}}<\frac{1}{\left|\sin\left(\omega_{f}\cdot \pi \right)\right|}$$

Therefore, the chip breaking condition of vibration drilling is affected by amplitude, feed, and frequency rotation ratio. Chip shape can be controlled by adjusting drilling process parameters.

**Figure 2:**
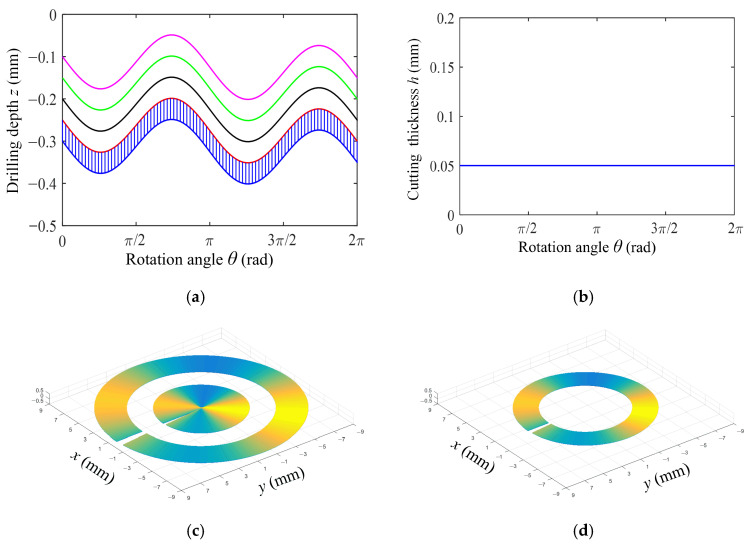
Axial motion track of the drill and three-dimensional shape of undeformed chips of each cutter tooth in one rotation period (feed *f*_r_ = 0.05 mm/r, amplitude *A* = 0.07 mm, frequency rotation ratio *ω_f_* = 2). (**a**) Tool axial motion path, (**b**) Under different drilling depths of drilling speed *n* = 1200 r/min and feed *f* = 0.08 mm/r, (**c**) Three-dimensional shape of undeformed chips of center and external cutter teeth, (**d**) Three-dimensional shape of undeformed chips with intermediate cutter teeth.

According to Equation (11), when the frequency rotation ratio *ω_f_* is an integer, the dynamic cutting thickness is always greater than zero. Figure 2 shows the change curve of the drill head’s axial movement track and the dynamic cutting thickness with the drill rotation angle when the frequency rotation ratio *ω_f_* is an integer of 2, and the shape of each cutter tooth without deformation.

Figure 2a,b show the axial motion trajectory and the dynamic theoretical cutting thickness of the tool when the frequency-to-rotation ratio is 2. The phase difference between two adjacent tool trajectory lines is π, with peaks corresponding to peaks and valleys corresponding to valleys. At this time, the tool has no idle cutting time, and the theoretical cutting thickness is the same as that of ordinary drilling. Figure 2c shows the three-dimensional morphology of undeformed chips on the inner and outer teeth of the BTA deep hole drilling tool, while Figure 2d shows the three-dimensional morphology of undeformed chips on the middle tooth of the BTA deep hole drilling tool. It can be seen that when the frequency rotation ratio is an integer, the axial motion trajectory of each rotation period of the drill is always disjoint, and the dynamic cutting thickness is equal to the feed amount *f*_r_.

When there is an intersection point between the axial movement track of the drill, there will be a moment when the dynamic cutting thickness is zero in the rotation cycle to achieve intermittent cutting, that is

(12) $$\frac{2A}{f_{r}}\geq \frac{1}{\left|\sin\left(\omega_{f}\cdot \pi \right)\right|}$$

**Figure 3:**
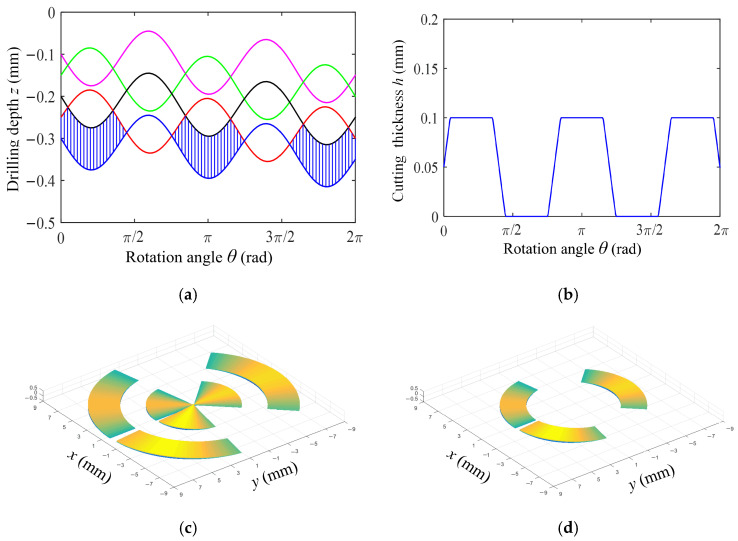
Axial motion track of drill bit and three-dimensional shape of undeformed chips of each cutter tooth in one rotation period (feed *f*_r_ = 0.05 mm/r, amplitude *A* = 0.07 mm, frequency rotation ratio *ω_f_* = 2.5). (**a**) Tool axial motion path, (**b**) Under different drilling depths of drilling speed *n* = 1200 r/min and feed *f* = 0.08 mm/r, (**c**) Three-dimensional shape of undeformed chips of center and external cutter teeth, (**d**) Three-dimensional shape of undeformed chips with intermediate cutter teeth.

In the actual drilling process, when the amplitude and the feed are constant, intermittent cutting can be achieved by changing the frequency rotation ratio, and different shapes of chips can be obtained. Figure 3 shows the variation curves of the axial movement track and dynamic cutting thickness with the drill rotation angle when the frequency rotation ratio is 2.5. It is noteworthy that the chips are in a discontinuous shape, and each complete unit chip is generated by 3 adjacent axial motion tracks and by 3 rotation cycles of the cutter teeth.

Figure 3a,b show the axial motion trajectory and dynamic theoretical cutting thickness of the tool when the frequency-to-rotation ratio is 2.5. The phase difference between two adjacent tool trajectory lines is π/2. Therefore, the phase difference between the first tool trajectory line and the third trajectory line is π, and the peak corresponds to the valley. At this time, the tool has the longest idle cutting time and the best chip-breaking effect. However, the impact at this time is also the greatest, as the theoretical cutting thickness is the largest. The instantaneous cutting thickness of the cutter teeth also changes periodically, and there will be a null cutting stage where the dynamic cutting thickness is zero. Figure 3c shows the three-dimensional morphology of the undeformed chips on the inner and outer teeth of the BTA deep hole drilling tool, and Figure 3d shows the three-dimensional morphology of the undeformed chips on the middle tooth of the BTA deep hole drilling tool.

Additionally, the three misaligned distribution cutter teeth for material removal, the BTA tool has two guide blocks distributed on the outer circumference of the body, which are the first guide block at an angle of approximately 90° clockwise from the cutting edge of the external cutter teeth and the second guide block at an angle of approximately 180° clockwise from the cutting edge of the external cutter teeth. In the process of BTA deep hole drilling, two guide blocks support and guide the drill. Through the elastic-plastic extrusion contact between the guide block and the hole wall, the radial and tangential component forces generated by the cutter teeth are balanced to ensure the self-centering of drilling and processing stability and realize the self-guiding in the process of deep hole machining to obtain better straightness. The first guide block is mainly used to balance the tangential force generated by the cutter teeth cutting. The second guide block is mainly used to balance the radial force generated by the cutter teeth cutting and controls the final size of the deep hole aperture together with the sizing edge of the external cutter teeth. The guide block balances the cutting force of the cutter teeth, while secondary plastic extrudes the hole wall formed by the cutter teeth cutting, which plays a good ironing effect.

**Figure 4:**
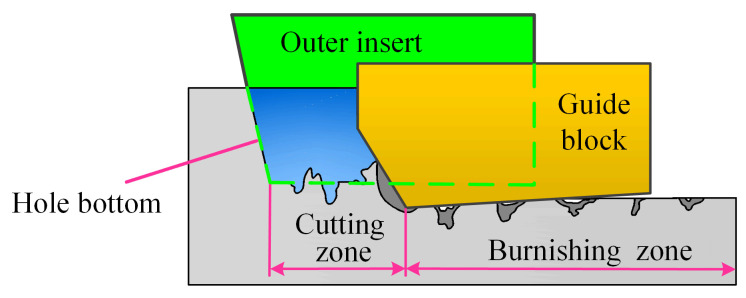
Formation process of surface texture of BTA deep hole machining.

The extrusion process of the guide block is illustrated in Figure 4. There are obvious differences between the cutting area of the sizing edge of the external cutter teeth and the extrusion deformation area of the guide block. Due to the tensile stress and tearing on the surface of the hole wall formed by the cutter teeth cutting, the hole wall area formed by the cutting edge of the external cutter teeth has an uneven surface topography of the peak and trough caused by the cutting residue, and the finish of the hole wall is poor [26].

When the guide block is squeezed into the cutting hole wall, due to the plastic flow of the metal workpiece material, the material will form a flow accumulation bump at the front end of the guide block, and the bump will be cut flat when it is in contact with the external cutter teeth sizing edge. At the same time, the two guide blocks will undergo strong squeezing deformation with the hole wall under the cutting force of the cutter teeth. When the squeezing force of the guide blocks on the hole wall reaches the yield limit, the hole wall will undergo plastic deformation. With the plastic flow of the material, the peak material in the squeezing area of the guide blocks will be filled into the valley. With the high-speed rotation and axial vibration of the drill, the hole wall will be subjected to multiple squeezing and trimming. The formation process of the surface texture of the hole wall drilled by axial vibration is complex.

## 3. Experimental Setup

**Figure 5:**
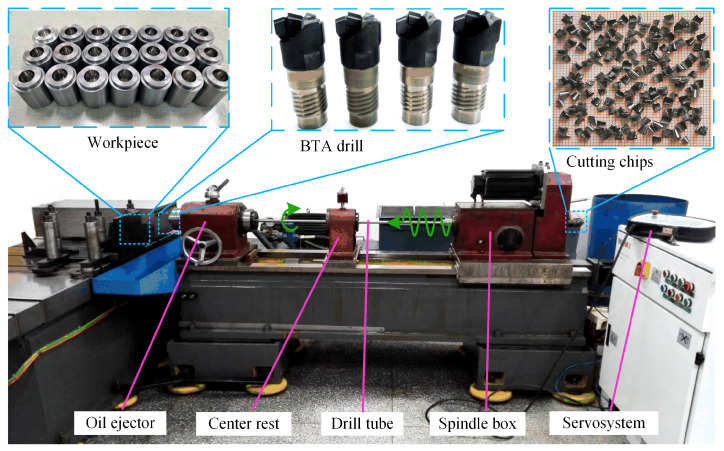
Tool rotary deep hole drilling machine with internal chip removal.

The experiment used a self-developed tool, a rotary internal chip removal deep hole drilling machine, which adopted an eccentric cam-type axial vibration-generating device. The experimental setup is shown in Figure 5. The main performance parameters of the machine are the maximum spindle speed of 2000 r/min, the position accuracy of the drill machine numerical control system is 0.001 mm, the flow rate of high-pressure cutting oil is 90 L/min, and the maximum pressure is 8 MPa. The experimental tool was a BTA deep hole drill with a Φ17.75 mm. The workpiece is Φ35 × 60 mm high-strength pressure vessel steel SA-508Gr3Cl2 (Tongxiang Metal Materials (Shanghai) Co., Ltd., Shanghai, China) (nuclear power steam generator heat exchange tube sheet substrate material); the element mass percentage of the workpiece material is shown in Table 1, and the mechanical property parameters are shown in Table 2. The drilling depth of each specimen was 50 mm. In order to ensure the reliability of the experimental results, experiments were conducted three times with each group of drilling parameters, and the average value of the measured results was taken as the final result. In this experiment, the TIME3230 contact surface roughness profilometer was used to measure the surface roughness of the hole wall. The measuring accuracy was 0.001 μm, the sampling length was 5 × 0.8 mm, and the evaluation length was 3 × 0.8 mm. Each specimen was measured at 3 different positions with a circumference interval of 90° from the hole wall, and each position was measured 4 times. The average value of the obtained was taken as the surface roughness value of the hole wall. At the same time, the specimen was cut from the direction parallel to the axis of the hole by wire cutting, and the microscopic morphology of the surface of the hole wall was photographed by the Olympus BX51 optical microscope.

## 4. Single Factor Experiment

The surface roughness of the BTA deep hole is influenced by process parameters such as drilling spindle speed *n*, drilling feed *f*_r_, vibration frequency *f*_w,_ and amplitude *A*. Firstly, a set of reference parameters: the drilling speed is 800 r/min, the drilling feed is 0.06 mm/r, the drilling amplitude is 0.14 mm, and the drilling vibration frequency is 27 Hz. Then, using the single-factor control variable method, the influence of vibration drilling process parameters on hole surface roughness is studied, and the results are compared and analyzed. The setting of process parameters of the single-factor experiment is shown in Table 3.

**Figure 6:**
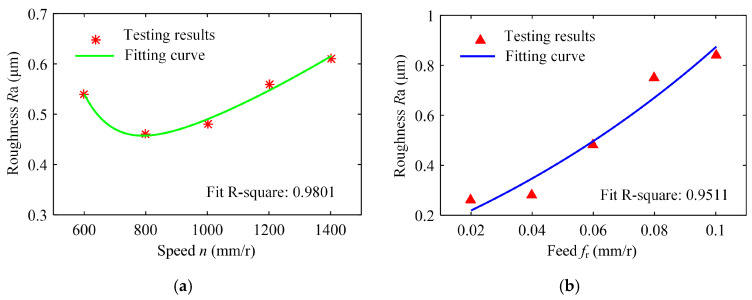

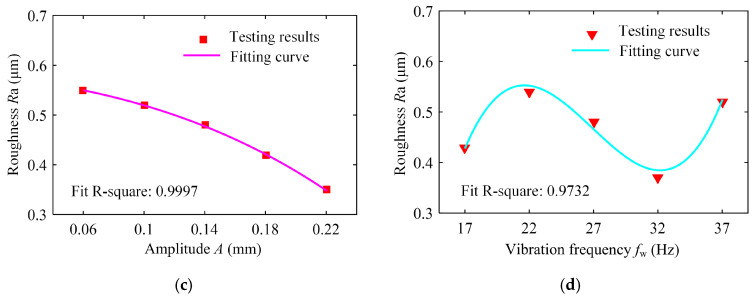
Influence of vibration drilling parameters on the surface roughness of the hole wall. (**a**) *f*_r_ = 0.06 mm/r, *A* = 0.14 mm, *f*_w_ = 27 Hz, (**b**) *n* = 800 r/min, *A* = 0.14 mm, *f*_w_ = 27 Hz, (**c**) *n* = 800 r/min, *f*_r_ = 0.06 mm/r, *f*_w_ = 27 Hz, (**d**) *n* = 800 r/min, *f*_r_ = 0.06 mm/r, *A* = 0.14 mm.

Under different vibration drilling process parameters, the drilling experiments were carried out on workpieces, and the changes of the surface roughness *R*a value of BTA deep hole drilling with drilling spindle speed, feed, amplitude, and vibration frequency were shown in Figure 6.

Figure 6a shows the variation of the surface roughness of the hole wall in vibration drilling with the spindle speed. Table 4 indicates the micro-morphology of the hole wall surface under variable speed conditions. When other conditions are certain, when the spindle speed is 800 r/min and 1000 r/min, the surface of the hole wall is smooth, and when the spindle speed rises to 1400 r/min, the surface of the knife mark is more obvious. When the spindle speed increased from 600 r/min to 800 r/min, the hole surface roughness *R*a decreased significantly, but when the spindle speed continued to increase, the surface roughness *R*a began to increase.

With the increase of drilling speed, the polishing effect of the guide block on the machined surface is enhanced, and the raised part of the machined surface is further polished and the groove is filled. However, as the speed is too large, the stability of the drilling system decreases, the vibration of the machine tool is too large, resulting in the surface finish of the hole wall decreasing and the surface roughness increasing. Therefore, the spindle speed should be appropriately increased to obtain better surface quality while ensuring the stability of the system.

The variation of the surface roughness of the hole wall in vibration drilling with the drilling feed is implied in Figure 6b, and the micro-morphology of the hole wall surface under variable drilling feed conditions is reported in Table 5. When other conditions are fixed, the microscopic quality of the hole wall surface gradually decreases with the increase of drilling feed. With the increase of drilling feed, the surface roughness value increases and the surface quality deteriorates obviously. Since the BTA deep hole drill will incline during drilling, the drilling force will increase with the increase of the feed, and the bending moment generated by the drilling tube to balance the drilling force will further increase the incline angle of the tool, resulting in poor surface quality.

The variation law of the surface roughness of the hole wall in vibration drilling with the drilling amplitude is revealed in Figure 6c, and the micro-morphology of the hole wall surface under the condition of varying amplitude is disclosed in Table 6. With the increase of drilling amplitude, the surface roughness of the hole wall decreases with the increase of drilling amplitude, and the surface micro-quality of the hole is obviously improved. When the amplitude increases, the energy of the tool is larger, the chip separation on the front tool surface is easier, and the defects on the machined surface are not easily formed. In addition, with the increase of drilling amplitude, the axial extrusion distance between the guide block and the hole wall increases, the ironing frequency of unit particles on the hole wall surface increases, and the quality of the hole wall is improved. However, the value of the amplitude cannot be too large, because the amplitude is too large to cause the instantaneous cutting force to rise sharply, making it easy to cause the edge to collapse and have an adverse effect on the stability of the drilling system.

The variation law of the surface roughness of the hole wall in vibration drilling with amplitude and frequency is exhibited in Figure 6d, and the micro-morphology of the hole wall surface under varying vibration frequency is shown in Table 7. The micro-morphologies of the pore surface obtained under different parameters are similar, and the effect of vibration frequency *f*_w_ on hole wall surface micro-morphologies is weak. The influence of vibration frequency on surface roughness is mainly reflected by the periodic change of frequency rotation ratio of 60*f*_w_/*n*. When frequency rotation ratio is close to an even number, the cutting thickness changes relatively gently, but it is constantly chipping. When the frequency ratio is close to odd, the cutting thickness changes sharply and the impact is serious, which is not conducive to the reduction of roughness. Therefore, the selection of parameters should take into account the influence of chip breaking and drilling process stability.

## 5. Modeling and Analysis

In the process of BTA deep hole axial vibration drilling, there are many factors affecting the surface roughness of the hole wall, so the BBD experimental design method is used to design the experimental plan. The Box–Behnken Design is a three-level design for adaptive response surface design proposed by Box and Behnken in 1960, which combines factor design with incomplete block design. Central composite design and BBD are commonly used second-order designs [30]. In contrast, the number of experiments designed by Box Behnken is usually less than that of the central composite design. Before implementing the BBD, factors are usually screened to eliminate unimportant ones. The advantage of the BBD method is that it estimates first-order, second-order, and interaction terms with fewer experiments and designs more economically and efficiently with the same number of factors, making it an effective response surface design method.

According to the single-factor experiment results and actual processing conditions, the appropriate BTA deep hole axial vibration drilling parameters were selected, and the BBD experiment with four factors and three levels was designed. The experimental factors and levels are shown in Table 8. The roughness experiment scheme and test results of BTA deep hole axial vibration drilling are shown in Table 9.

Response surface methodology (RSM) is a modeling method used to establish statistical or mathematical relationships between input variables and responses [30]. Derived from mathematical and statistical techniques, this method uses the least square method to solve the regression coefficient of the model, improvement, and optimization of the process. The prediction model of the hole wall surface roughness of BTA deep hole machining is established by RSM. The second-order polynomial model is as

(13) $$y=\beta_{0}+\sum_{i=1}^{k}\beta_{i}x_{i}+\sum_{i=1}^{i}\beta_{ii}{x_{i}}^{2}+\sum_{i}\sum_{j}\beta_{ij}x_{i}x_{j}+\varepsilon ,\;\;\;i<j$$

where *y* is the response variable, *x_i_* is the input variable, *β_i_* is the coefficient of the linear term, *β_ii_* is the coefficient of the square term, *β_ij_* is the coefficient of the interaction term, *k* is the number of input variables, and *ε* is the fitting error or residual.

Design Expert software was used to perform second-order regression fitting for hole wall surface roughness combined with BBD experimental measurement results exhibited in Table 9. The second-order prediction model of hole wall surface roughness for BTA deep hole axial vibration drilling was obtained, as shown in Equation (14), and the fitting results of factor coefficients were explained in Table 10.

(14) $$\begin{array}{l} Ra=\beta_{0}+\beta_{1}n+\beta_{2}f_{r}+\beta_{3}f_{w}+\beta_{4}A+\beta_{12}(n\cdot f_{r})+\beta_{13}(n\cdot f_{w})+\beta_{14}(n\cdot A)\\ +\beta_{23}(f_{r}\cdot f_{w})+\beta_{24}(f_{r}\cdot A)+\beta_{34}(f_{w}\cdot A)+\beta_{11}(n^{2})+\beta_{22}({f_{r}}^{2})+\beta_{33}({f_{w}}^{2})+\beta_{44}(A^{2}) \end{array}$$

**Figure 7:**
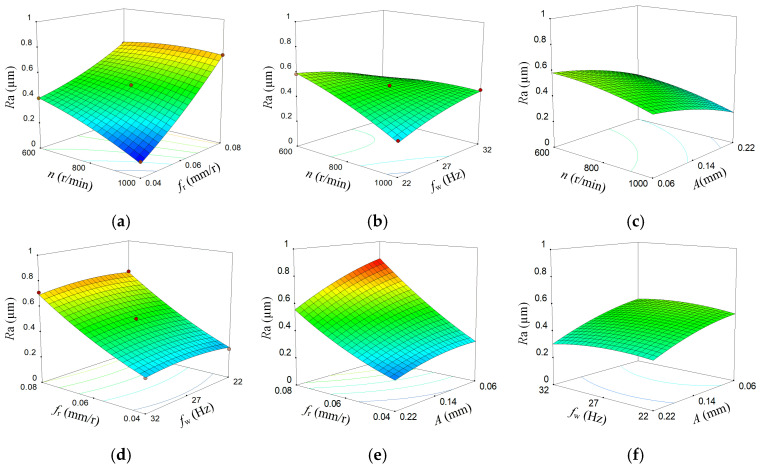
3D response surface of hole wall roughness. (**a**) The effect of speed and feed, (**b**) The effect of speed and frequency, (**c**) The effect of speed and amplitude, (**d**) The effect of feed and frequency, (**e**) The effect of feed and amplitude, (**f**) The effect of frequency and amplitude.

In order to further reveal the influence of BTA deep hole vibration drilling process parameter factors and their interaction on hole wall surface roughness, the three-dimensional response surface roughness corresponding to the other two factors was obtained by keeping two of the four process parameters at 0 level, and the results are shown in Figure 7.

Figure 7a shows the effect of speed and feed. As the spindle speed n increases, the surface roughness *R*a of the hole gradually decreases. As the feed *f*_r_ increases, the surface roughness *R*a of the hole wall also increases. Figure 7b shows the effect of speed and frequency. As the speed increases, the roughness decreases, and as the drilling vibration frequency increases, the roughness increases. Figure 7c shows the effect of speed and amplitude. As the speed increases, the roughness decreases, and as the drilling amplitude increases, the roughness decreases. Figure 7d shows the effect of feed and vibration frequency. As the feed increases, the roughness also increases, but the effect of vibration frequency is not significant. Figure 7e shows the effect of feed and amplitude. As the feed increases, the roughness increases, and as the amplitude increases, the roughness decreases. Figure 7f shows the effect of vibration frequency and amplitude. As the vibration frequency increases, the surface roughness first increases and then slightly decreases, but its range of variation is small and the degree of influence is weak. As the amplitude increases, the roughness also increases.

The results of variance analysis of hole wall surface roughness are shown in Table 11. Analysis of Variance (ANOVA) is a tool widely used in model significance tests. Its essence is to compare the change of response value caused by input factors with the change of response value caused by experimental random error, so as to determine whether the influence of factors on the response value is significant. In the ANOVA tables, the total sum of squares of deviation (SS) reflects the difference between each measurement and the mean. The *p*-value reflects the confidence level of the model; *p* < 0.05 means that the corresponding factor has a significant effect on the response value, and *p* < 0.0001 indicates that the effect is extremely significant.

It can be seen that the overall *p*-value of the model is less than 0.0001, indicating that the second-order response model of surface roughness constructed is significant. Spindle speed *n*, feed *f*_r_, amplitude *A*, the interaction term *n*-*f*_r_ between spindle speed and feed, and the interaction term *n*-*f*_w_ between spindle speed and vibration frequency have extremely significant effects on the surface roughness of the hole wall (*p* < 0.0001). The interaction between feed rate and amplitude *f*_r_-*A*, as well as the square term of each process parameter (*n*^2^, *f*_r_^2^, *f*_w_^2^, *A*^2^) has a significant effect on the surface roughness of the hole wall (0.0001 < *p* < 0.05). According to the F value of variance analysis, the significant order of influence of each process parameter on the surface roughness of vibration-drilled holes is as follows: drilling feed *f*_r_, amplitude *A*, spindle speed *n*, and vibration frequency *f*_w_.

**Figure 8:**
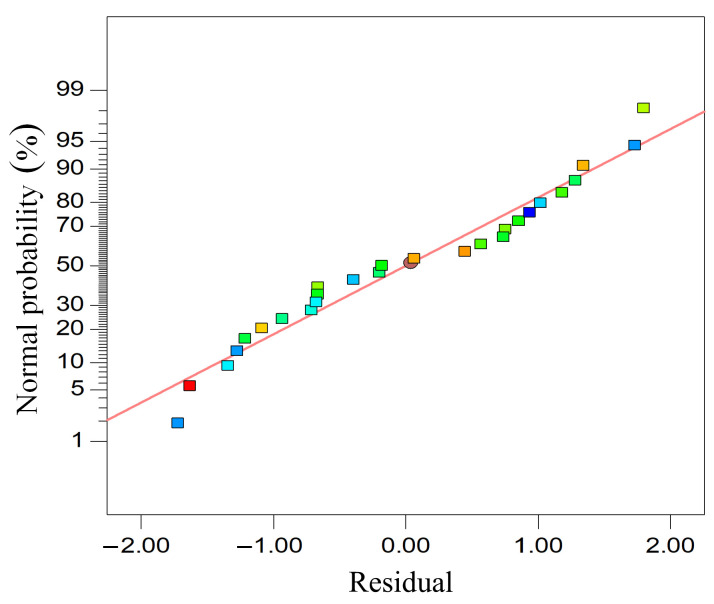
Normal probability of residual surface roughness.

The complex correlation coefficient R^2^ = 0.9948 indicates that the predicted value of the model has a good agreement with the measured value. The correction correlation coefficient Adj R^2^ = 0.9888 indicates that the model can explain 99.48% of the variation of response value. The residual normal probability of the surface roughness prediction model is shown in Figure 8. It can be seen that there is a straight line with an inclination angle of 45°, which represents the predicted surface roughness values under different experimental conditions. The box represents the experimental value; theoretically, all experimental values should be on this straight line. There is a certain deviation between the experimental values and the theoretical predicted values, but most of the experimental values are very close to the theoretical straight line, indicating that the proposed prediction model can better reflect the real data situation. The residual handicap is distributed near the normal distribution line, indicating that there is no large deviation in the data and the model is well fitted.

## 6. Optimization and Discussion

The satisfaction function method is a method used to deal with response optimization problems. In this method, each response value y is converted to full intension d by the satisfaction function, and its value range is 0 ≤ *d* ≤ 1. The closer the satisfaction degree *d* is to 1, the closer the response value is to the target value [32]. All input parameters are limited within the corresponding limit range. After calculating and analyzing the single-objective optimization mathematical model using design expert software, the drilling parameter combination for minimizing the surface roughness *R*a is obtained. In this paper, the hole wall surface roughness *R*a is minimized as the optimization objective, and the satisfaction function as shown below is established as

(15) $$d=\left\{\begin{array}{l} \;\;\;\;\;\;\;\;\;\;\;\;\;\;\;\;\;1\;\;\;\;\;\;\;\;\;\;\;\;\;\;\;\;\;\;\;\;\;\;\;\;R_{a}\leq L\\ {\left|\frac{R_{a}(n,f_{r},A,f_{w})-U}{L-U}\right|}^{q}\;\;\;L\leq R_{a}\leq U\\ \;\;\;\;\;\;\;\;\;\;\;\;\;\;\;\;\;0\;\;\;\;\;\;\;\;\;\;\;\;\;\;\;\;\;\;\;\;\;\;\;\;R_{a}\geq U \end{array}\right.$$

where, *q* is the weight value; *L* and *U* are the set values of the upper limit and lower limit of the surface roughness of the hole wall, respectively.

**Figure 9:**
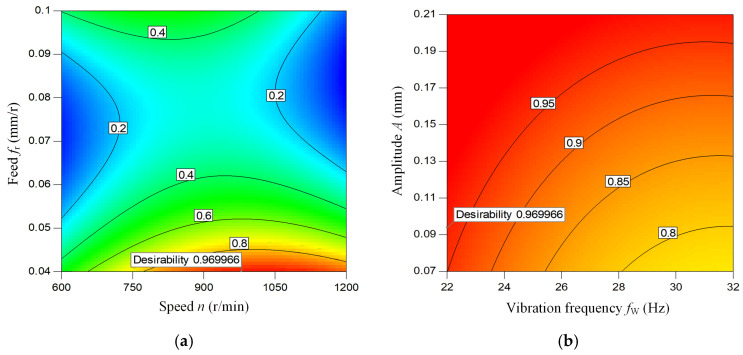
Contour lines of surface roughness satisfaction function. (**a**) The effect of speed and feed, (**b**) The effect of amplitude and vibration frequency.

Based on the actual processing conditions of BTA deep hole vibration drilling, the constraints *L* and *U* used for optimization are 0.08 µm and 0.9 µm, respectively. On the basis of the prediction model of hole wall surface roughness for BTA deep hole vibration drilling, the satisfaction function method was used to optimize the hole wall surface roughness value. The contour lines of the corresponding satisfaction function are shown in Figure 9, and the maximum satisfaction *d* of the surface roughness is 0.969.

According to the satisfaction function method, the surface roughness of BTA deep hole axial vibration drilling was optimized, and the optimal drilling process parameters were obtained as follows: spindle speed *n* = 953.8 r/min, feed rate *f*_r_ = 0.04 mm/r, amplitude *A* = 0.094 mm, vibration frequency *f*_w_ = 28 Hz. The optimal value of the corresponding surface roughness of the drilling hole wall *R*a = 0.095 µm.

**Figure 10:**
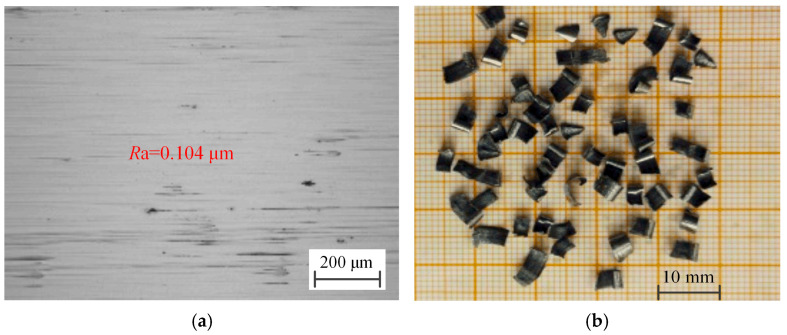
Micro-morphology of hole wall surface drilled and formed chips with optimized process parameters. (**a**) Micro-morphology of hole wall surface, (**b**) formed chip in BTA deep hole drilling with vibration assistance.

The optimal drilling process parameters were used for several experiments, and the average surface roughness *R*a of the hole wall was measured to be 0.104 µm, and the micro-morphology of the hole wall surface drilled and formed chips with optimized process parameters was shown in Figure 10. Compared with the experimental values, the error of the established model is 8.65%, which indicates that the established model can accurately predict the surface roughness of BTA deep holes by axial vibration drilling.

The chips formed by BTA deep hole vibration drilling using the optimal drilling process parameters are shown in Figure 10b. Due to the good impact effect of vibration drilling, the continuous cutting of the three cutter teeth of the BTA deep hole drilling and the workpiece material becomes the interrupted cutting. There is a zero-cutting thickness of the airspace, which has a good chip-breaking effect, can make the chip into the ideal small shape, the external cutter teeth and the center cutter of the formation of small chips into the drill chip mouth, the intermediate cutter teeth into the small chip mouth, and finally discharged from the tube cavity. The good chip-breaking effect ensures the continuous and smooth BTA deep hole machining.

**Figure 11:**
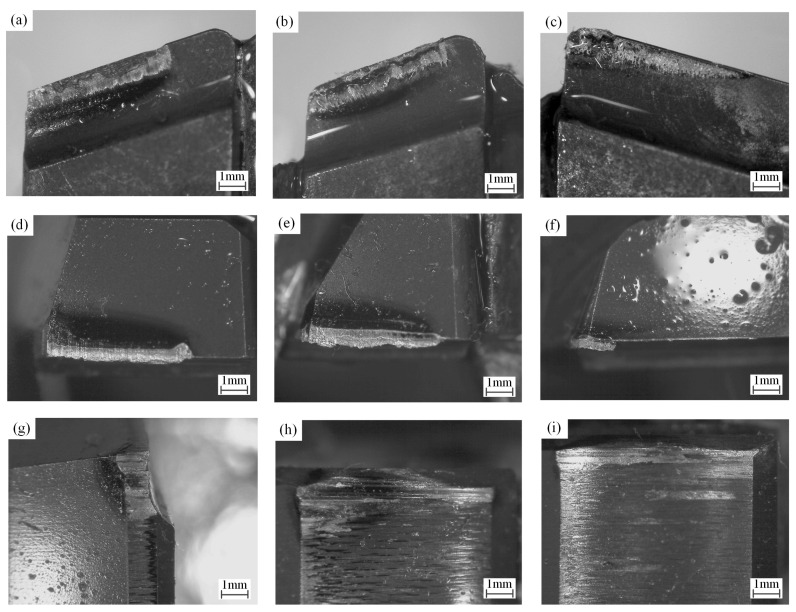
Wear morphology of BTA deep hole drilling with vibration assistance. (**a**) The rake face of external cutter teeth, (**b**) the rake face of the intermediate cutter teeth, (**c**) the rake face of the center cutter teeth, (**d**) the flank of external cutter teeth, (**e**) the flank of intermediate cutter teeth, (**f**) the flank of center cutter teeth, (**g**) the edge sizing blade of the external cutter teeth, (**h**) the first guide block, (**i**) the second guide block.

Using the optimal drilling process parameters, BTA deep hole vibration drilling SA-508Gr3Cl2 specimens of nuclear power steam generator heat exchange tube sheet substrate material, the drilling depth of each hole is 800 mm. After drilling 10 holes, the wear pattern of the BTA bit after the cumulative drilling length of 8000 mm is shown in Figure 11.

The main wear area of the BTA drilling tool is mainly divided into 9 parts, which are the rake face and flank of the three cutter teeth, the edge sizing blade of the external cutter teeth, the first guide block, and the second guide block. The rake face of the three cutter teeth mainly interacts with the chip, the flank of the three cutter teeth mainly interacts with the hole wall, and the edge-sizing blade of the external cutter teeth and the two guide blocks all interact with the hole wall. Since the sizing edge and guide block of the drill are designed with a wedge cone angle, the edge sizing blade and guide block are all in contact with the hole wall at the top area of the front end of the drill. Due to the reciprocating axial movement of the bit during vibration drilling, the high-pressure cutting oil is easy to reach the high-pressure contact area between the cutter teeth and the workpiece material cutting and the guide block and the hole wall extrusion, which plays a role in lubricating, reducing friction, and reducing the drilling temperature, resulting in the reduction of the tool wear. Vibration drilling can reduce BTA drill wear and improve tool life.

## 7. Conclusions

By experimental investigation and modeling of surface roughness in BTA deep hole drilling with vibration assistance, the following conclusions were obtained.

The mathematical equation of large amplitude and low frequency motion of the BTA deep hole vibration drilling tool is established, the chip breaking mechanism of the three cutter teeth of the BTA deep hole vibration drilling is analyzed, and the chip length control method is given. The influencing factors of hole wall forming under the vibration drilling condition are explored.

The effect of vibration drilling parameters on the surface roughness was studied by a single-factor experiment. With the increase of drilling speed, the surface roughness of the hole wall decreases first and then increases. The surface roughness increases with the increase of drilling feed but decreases with the increase of amplitude. The effect of the vibration frequency on hole surface roughness is relatively weak.

On the basis of the BBD experiment, a second-order empirical model for predicting hole surface roughness is established. The accuracy of the prediction model was analyzed using ANOVA, and the complex correlation coefficient of the model was 0.9948. The model can well reflect the mapping relationship between input parameters and surface roughness. According to the F-value analysis, the order of the significant influence of process parameters on the surface roughness of the hole is as follows: drilling feed *f*_r_, amplitude *A*, speed *n*, and vibration frequency *f*_w_.

Based on the established second-order response model of surface roughness, the machining process parameters are optimized using the satisfaction function method. Through optimization analysis and experimental verification, the obtained vibration drilling parameters can achieve a smaller surface roughness of the hole wall. Vibration drilling can reduce the wear of the BTA drill and improve tool life. The error between the predicted value of the established model and the experimental measurement value is 8.65%, indicating that the established prediction model is reliable and can accurately predict the surface roughness of the hole wall for the BTA deep hole axial vibration drilling. This method has been validated through experiments and can be used for academic research and industrial production.

## Figures and Tables

**Table 1:** Element mass percentage of SA-508Gr3Cl2.

Material	Mn	Cr	Si	Al	Ni	C	S	P	V	Mo	Fe
SA-508Gr3Cl2	1.4	0.21	0.32	0.02	0.75	0.16	0.015	0.015	0.03	0.052	other

**Table 2:** Mechanical characteristic parameters of SA-508Gr3Cl2.

Material	Elasticity Modulus *E* (GPa)	Yield Strength σ_s_ (MPa)	Tensile Strength *σ*_b_ (MPa)	Poisson Ratio *ε*	Elongation *δ*	Reduction of Area *ψ*	Density *ρ* (Kg/m^3^)
SA-508Gr3Cl2	210	620	740	0.269	24%	40%	7920

**Table 3:** Parameter setting of single factor test.

Parameter	Parameter Levels	Reference Value
Speed *n* (r/min)	600, 800, 1000, 1200, 1400	800
Feed *f*_r_ (mm/r)	0.02, 0.04, 0.06, 0.08, 0.10	0.06
Amplitude *A* (mm)	0.06, 0.10, 0.14, 0.18, 0.22	0.14
Vibration frequency *f*_w_ (Hz)	17, 22, 27, 32, 37	27

**Table 4:** Micro-morphology of the hole wall surface under variable speed conditions.

Speed *n* (r/min)	600	1000	1400
Microscopic morphology (Roughness)	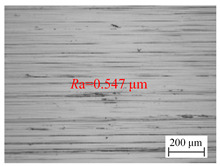	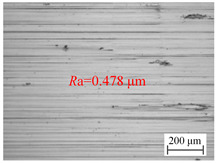	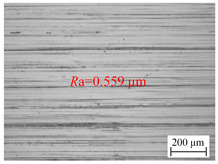

**Table 5:** Micro-morphology of the hole wall surface under variable feed conditions.

Feed *f*_r_ (mm/r)	0.02	0.06	0.1
Microscopic morphology (Roughness)	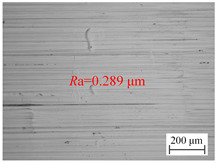	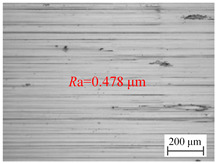	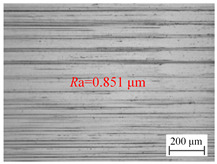

**Table 6:** Micro-morphology of the hole wall surface under variable amplitude condition.

Amplitude *A* (mm)	0.06	0.14	0.22
Microscopic morphology (Roughness)	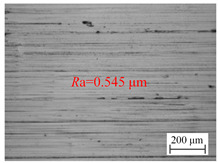	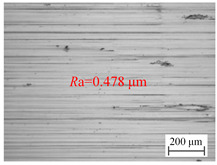	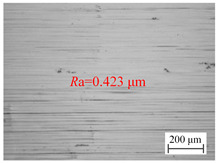

**Table 7:** Micro-morphology of the hole wall surface under variable vibration frequency condition.

Vibration Frequency *f*_w_ (Hz)	17	27	37
Microscopic morphology (Roughness)	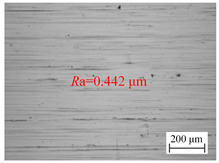	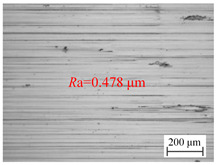	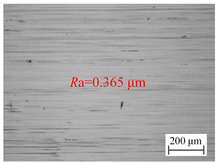

**Table 8:** Factors and levels of BBD experiment.

Factors	Levels		
	−1	0	1
Speed *n* (r/min)	600	800	1000
Feed *f*_r_ (mm/r)	0.04	0.06	0.08
Amplitude *A* (mm)	0.07	0.14	0.21
Vibration frequency *f*_w_ (Hz)	22	27	32

**Table 9:** BBD experimental scheme and experimental results.

Test No.	Speed *n* (r/min)	Feed *f*_r_ (mm/r)	Amplitud *A* (mm)	Vibration Frequency *f*_w_ (Hz)	Roughness *R*a (μm)
1	800	0.06	0.22	32	0.292
2	800	0.06	0.14	27	0.495
3	600	0.08	0.14	27	0.695
4	600	0.06	0.06	27	0.611
5	1000	0.06	0.14	22	0.271
6	800	0.04	0.14	32	0.26
7	600	0.04	0.14	27	0.405
8	800	0.08	0.06	27	0.843
9	800	0.08	0.22	27	0.572
10	800	0.08	0.14	32	0.715
11	1000	0.06	0.06	27	0.439
12	1000	0.08	0.14	27	0.72
13	800	0.06	0.22	22	0.329
14	800	0.04	0.14	22	0.233
15	800	0.04	0.06	27	0.295
16	800	0.06	0.14	27	0.47
17	600	0.06	0.14	22	0.588
18	800	0.06	0.06	22	0.528
19	1000	0.06	0.22	27	0.227
20	600	0.06	0.22	27	0.417
21	800	0.06	0.14	27	0.478
22	800	0.06	0.06	32	0.537
23	800	0.04	0.22	27	0.229
24	1000	0.06	0.14	32	0.455
25	1000	0.04	0.14	27	0.12
26	800	0.08	0.14	22	0.734
27	600	0.06	0.14	32	0.384

**Table 10:** Coefficients of the surface roughness response surface model.

Coefficient	*β*_0_	*β*_1_	*β*_2_	*β*_3_	*β*_4_	*β*_12_	*β*_13_	*β*_14_
Value	1.621	−2.979 × 10^−3^	−6.406	−4.972 × 10^−3^	2.988	1.938 × 10^−2^	9.700 × 10^−5^	−2.813 × 10^−4^
Coefficient	*β*_23_	*β*_24_	*β*_34_	*β*_11_	*β*_22_	*β*_33_	*β*_44_	
Value	−0.115	−32.031	−2.875 × 10^−2^	−7.031 × 10^−7^	82.500	−1.155 × 10^−3^	−4.648	

**Table 11:** Results of variance analysis of hole wall surface roughness.

Source of Variance	Sum of Squares	Degree of Freedom	Mean Square	F Value	*p* Value	Remarks
Model	0.90	14	0.064	164.48	<0.0001	significant
*n*	0.063	1	0.063	160.14	<0.0001	significant
*f*_r_	0.62	1	0.62	1592.20	<0.0001	significant
*f*_w_	1.333 × 10^−4^	1	1.333 × 10^−4^	0.34	0.5706	Not significant
*A*	0.12	1	0.12	299.47	<0.0001	significant
*n*-*f*_r_	0.024	1	0.024	61.28	<0.0001	significant
*n*-*f*_w_	0.038	1	0.038	95.99	<0.0001	significant
*n*-*A*	8.100 × 10^−5^	1	8.100 × 10^−5^	0.21	0.6576	Not significant
*f*_r_-*f*_w_	5.290 × 10^−4^	1	5.290 × 10^−4^	1.35	0.2680	Not significant
*f*_r_-*A*	0.011	1	0.011	26.80	0.0002	significant
*f*_w_-*A*	5.290 × 10^−4^	1	5.290 × 10^−4^	1.35	0.2680	Not significant
*n* ^2^	4.219 × 10^−3^	1	4.219 × 10^−3^	10.76	0.0066	significant
*f*_r_^2^	5.808 × 10^−3^	1	5.808 × 10^−3^	14.81	0.0023	significant
*f*_w_^2^	4.447 × 10^−3^	1	4.447 × 10^−3^	11.34	0.0056	significant
*A* ^2^	4.720 × 10^−3^	1	4.720 × 10^−3^	12.04	0.0046	significant
Residual	4.705 × 10^−3^	12	3.921 × 10^−4^			
Lack of fit	4.379 × 10^−3^	10	4.379 × 10^−4^	2.69	0.3016	Not significant
Error term	3.260 × 10^−4^	2	1.630 × 10^−4^			
Total	0.91	26				
Mean = 0.46 μm; R^2^ = 0.9948; Adj R^2^ = 0.9888; Pred R^2^ = 0.9714						

## Data Availability

Data are contained within the article.
